# Fulfilling end-of-life dreams: A scoping review of bucket lists in palliative and hospice care

**DOI:** 10.1017/S1478951525100473

**Published:** 2025-09-12

**Authors:** Swasati Handique, Michael Bennett, Scott D Ryan

**Affiliations:** 1 School of Social Work, The University of Texas at Arlington, Arlington, TX, USA; 2 School of Social Work, University of Windsor, Windsor, ON, Canada

**Keywords:** Bucket list, wish fulfillment, dreams, palliative care, hospice care

## Abstract

**Objectives:**

The purpose of this study is to examine the existing literature on end-of-life dream experiences and bucket list fulfillments among terminally ill individuals receiving hospice and/or palliative care.

**Methods:**

A scoping review registered on Open Science Framework was conducted in accordance with the guidelines for Scoping reviews (PRISMA-ScR). An electronic search of literature was generated from EBSCO databases published until June 2024. Studies were included if they described and evaluated the effects of bucket lists and/or end-of-life wish fulfillment.

**Results:**

This review identified 2,234 studies, and 11 of these were included in the review. Four major themes were established using thematic content analysis: (1) impact on holistic well-being, (2) role of family in wish fulfillment, (3) cultivation of gratitude, and (4) collaborative leadership in wish fulfillment. In wish fulfillment, the results significantly pointed to the need for more intricate evaluation among patients and interventions that cover beyond the physical aspect.

**Significance of results:**

Palliative and hospice care settings should work toward securing sustainable funding for structured wish-fulfillment programs to address existing accessibility gaps and further enhance the holistic nature of care in these settings. Wish-fulfillment interventions represent a powerful tool in enhancing dignity and holistic experiences for terminally ill patients. Future research efforts could strengthen programs ensuring every individual is able to achieve a sense of peace, fulfillment, and closure during their care trajectory.

## Introduction

Palliative and hospice care are essential components of end-of-life support, offering assistance and solace to individuals in the final stages of life as well as to their loved ones. Whereas curative care focuses on interventions that aim to save lives, palliative care is a comprehensive approach to care that strives to meet the holistic needs of individuals who are living with serious, long-term illness (Clark et al. 2015; Omilion-Hodges and Swords 2017; Thomas et al. 2018). Hospice care, by extension, prioritizes providing compassionate and high-quality care to individuals with terminal illness, enabling them to live their optimal quality of life (Rhymes 1990; Saunders 1978). The ideal palliative care approach integrates medical treatments with psychosocial, emotional, and spiritual interventions, aiming to deliver comfort and dignity throughout the dying process (Egan et al. 2010). The individuals and their families who are receiving palliative care benefit from compassionate care and practical and psychosocial assistance (Gómez-Batiste and Connor 2017; Swerissen and Duckett 2014).

Contemporary models of hospice palliative care emphasize the importance of extending beyond symptom and pain control and remaining focused on effective symptom management and psychosocial and spiritual interventions. Given its holistic nature, it is also important to consider an individual’s end-of-life goals or personal aspirations, which are the cornerstones of end-of-life care (Merluzzi et al. 2024). Indeed, if goals of care conversations are truly centered on an individual’s needs, they must also include discussions about hopes, dreams, and aspirations. In this sense, a last wish in palliative care can be characterized as a felt or expressed desire not limited to the medical domain, which holds personal significance in an individual’s final phase of life (Back et al. 2014). Similarly, like having a last wish or personal wish, a bucket list in general is understood as a “set of meaningful goals that a person hopes to achieve before they die” (Chu et al. 2018, p. 151). Fulfilling a last wish or accomplishing a “bucket list” desire can serve as an intervention to assist in preparing for an anticipated death (Delgado et al. 2016).

The term “bucket list” originates from the phrase “kicking the bucket,” which refers to a list of things that a person wants to achieve before dying (Webster 2018). The popularity of the term bucket list rose with the release of the film *The Bucket List*, sparking discussions about individual desires before dying (Reiner 2008). While “bucket list” is not an official medical term, the process of creating one is well recognized. Acknowledging and honoring last wishes can enhance a person’s sense of control, affirm their individuality, provide effective symptom management, facilitate decision-making, and prepare them for a peaceful passing. The fulfillment of a wish can instill hope, affirm the value that life still has even when limited in time and potential, and recapture the essence of enjoying life (Granger 2012). Fulfilling a last wish can serve as an end-of-life intervention focused on preparing for the approaching death, offering an opportunity, even if temporary, to experience a sense of wholeness in physical, emotional, and spiritual aspects (Ferrell and Coyle 2002; Institute of Medicine (US) 2003).

By discussing an individual’s last wishes or bucket list, care providers can tailor their support accordingly and use these personal wishes as a guide for evaluating the care provided (Brinkman et al. 2014; Sudore et al. 2017). These conversations allow individuals to express their desires and participate in their end-of-life care (Raisio et al. 2015). Indeed, research has demonstrated that participation in advance care planning can enhance the quality of end-of-life care (Brinkman et al. 2014; Steinhauser et al. 2015). However, past research has been more focused on the importance of goals of care conversations as a vital part of advance care planning for individuals receiving palliative and/or end-of-life care (Childers et al. 2017; Roze Des Ordons et al. 2016; You et al. 2014) with discussions predominantly around person-centered, treatment preferences and end-of-life treatments (González-González et al. 2020; Kuosmanen et al. 2021; Leng et al. 2022). These approaches tend to overlook the individuals’ desired milestones and how they wish to live in their remaining time. Without these discussions, it may be challenging to provide person-centered care, as views and preferences may differ among individuals, their loved ones, and healthcare personnel (Malhotra et al. 2015; Steinhauser et al. 2015). Curating a bucket list or discussing one’s personal last wish is a way for individuals to identify what they want for themselves and what matters most to them. Scant literature exists on bucket list conversations in end-of-life care, and their impact on the overall care experience remains unclear. Thus, the purpose of this scoping review is to examine the existing literature on end-of-life dream experiences and bucket list fulfillments among terminally ill individuals receiving hospice and/or palliative care. This study aims to enhance understanding of how fulfilling end-of-life dreams and bucket list wishes can enrich service users’ quality of life and influence their care experience.

## Methods

A scoping review is defined as “a form of knowledge synthesis that addresses an exploratory research question aimed at mapping key concepts, types of evidence, and gaps in research related to a defined area or field by systematically searching, selecting, and synthesizing existing knowledge” (Colquhoun et al. 2014, p. 1292). This scoping review was conducted between June and October 2024 in accordance with Arksey and O’Malley’s (2005) 5 stage framework: (1) identifying the research question, (2) identifying relevant studies, (3) selecting the studies, (4) charting the data, and (5) collating, summarizing, and reporting the results. As this study was an analysis of extant published literature, no approval was required from the university’s institutional review board to conduct this scoping review. In addition, this scoping review was conducted using Preferred Reporting Items for Systematic Reviews and Meta-Analyses extension for Scoping Reviews (PRISMA-ScR) to show the number of publications identified and screened for eligibility during the scoping review process (Arksey and O’Malley 2005; McGowan et al. 2020). The scoping review included all scholarly (e.g., peer-reviewed) research studies published from January 1, 2000, through June 2024. Protocol for this study can be accessed at osf.io/nw6m9.

### Identifying the research question

This review was guided by the research question: *What is the state of the literature about end-of-life dream experiences/bucket list fulfillment among terminally ill patients receiving hospice and/or palliative care?*

### Identifying relevant studies

**Figure 1.**
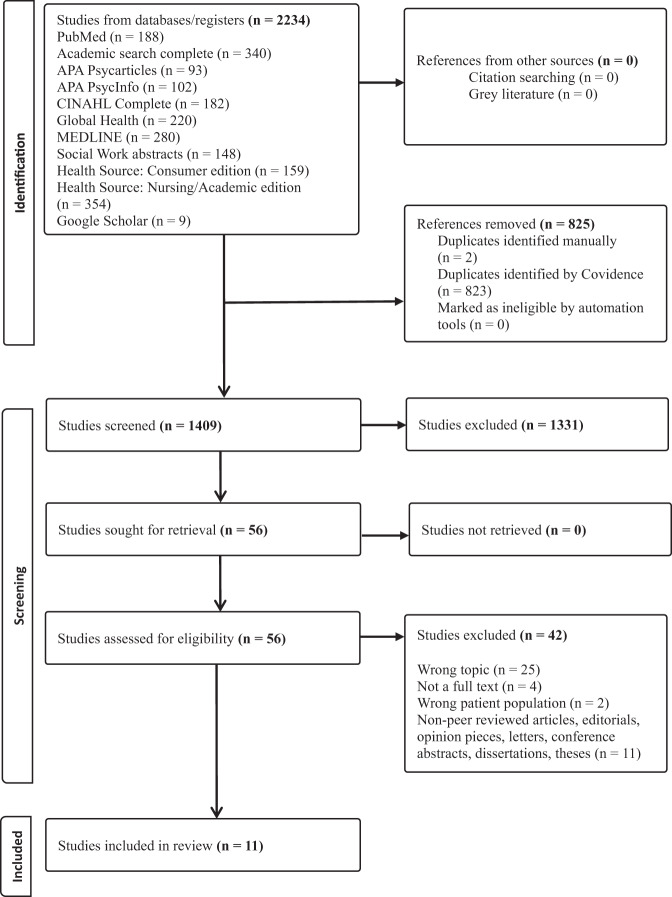
PRISMA chart.

A detailed flow chart of the literature search process is provided in Fig. 1. This review utilized electronic databases to identify potentially relevant studies in the EBSCO platform to search relevant studies from various disciplines: PubMed, Academic Search Complete, APA PsycArticles, APA PsycInfo, CINAHL Complete, Global Health, MEDLINE, Social Work Abstracts, Health Source – Consumer Edition, and Health Source: Nursing/Academic Edition. The search terms and medical subject headings (MeSH) included (“end-of-life care” OR “end of life care” OR “terminal care” OR “terminally ill” OR “terminal illness”) AND (“Make-a-wish” “children’s wish” and “dream foundation”) AND (“bucket list” OR “bucket list fulfil*” OR “wish” OR “wish fulfil*” OR “dreams” OR “last dream” OR “end-of-life dream” OR “life aspiration” OR “unfinished business” OR “unresolved matters” OR “unfulfilled desires” OR “unachieved goals”) AND (“hospice” OR “palliative care”) NOT (sleep OR vision*) were used, along with various combinations with Boolean operators (e.g., AND, OR, NOT) when searching for peer-reviewed scholarly articles.

### Study selection

Inclusion and exclusion criteria were operationalized in the following way. To exclude studies that did not address the research question, the reviewers applied inclusion criteria to the articles obtained from both the databases and reference list searches (Arksey and O’Malley 2005). The inclusion criteria for this study were (all of which must be met): (1) studies involving terminally ill patients receiving hospice/palliative care; (2) studies examining last wish experiences and/or bucket lists fulfillment; (3) studies focusing on interventions or programs aimed at fulfilling bucket list items or addressing last wish experiences; (4) studies reporting on the impact or outcomes of last wish experiences or bucket list fulfillment on individuals’ well-being, quality of life, psychological state, or similar measures; (5) qualitative, quantitative, and mixed-methods studies; (6) peer-reviewed articles, including case studies, cohort studies, cross-sectional studies, RCTs, and systematic reviews; (7) studies published from the year 2000 onward; and (8) studies published in English. For the scope of this paper, bucket list is being defined as an individual’s values, identity, and desire for fulfillment beyond clinical considerations. Bucket list items may include travel, personal achievements, reconnections with loved ones, creative pursuits, or other life-enriching experiences that contribute to a sense of meaning and legacy in the face of a life-limiting illness.

The exclusion criteria were (1) studies involving patients who are not terminally ill; (2) studies not examining last wish experiences/bucket list fulfillment; (3) studies focusing on general palliative care interventions without specific mention of last wish experiences or bucket list fulfillment; (4) studies that do not report relevant outcomes related to last wish experiences or bucket list fulfillment; and (5) non-peer-reviewed articles, review studies, editorials, opinion pieces, letters, and conference abstracts.

Covidence is a web-based systematic and scoping review reference management program used in this study to to screen the articles. During the title and abstract screening phase, one researcher independently reviewed the articles according to the inclusion and exclusion criteria, excluding those that did not meet the requirements. For the full-text analysis, two researchers independently screened the studies in Covidence, with a third researcher resolving any conflicts through discussion until consensus was reached.

### Charting the data

According to Arksey and O’Malley (2005), charting is a “technique for synthesizing and interpreting qualitative data by sifting, charting, and sorting material according to key issues and themes” (p. 26). Additionally, the PRISMA-ScR checklist requires researchers to individually report the characteristics of each selected article (McGowan et al. 2020). Thus, a data-charting table was created (Table 1), which consists of a mixture of general information: authors, year of publication, study location, population and sample size, methods, aim/purpose, theoretical/conceptual framework, key concepts of how the study defines/operationalizes “bucket lists” or “dream experiences” measures and outcomes, and key findings, implications, and recommendations.

**Table 1.** Data chart

Authors	Title of the study	Country of origin	Study population	Purpose of the study	Research design	Outcome measure	Key findings
Periyakoil et al. (2018)	Common items on a bucket list	United States	3,056 participants: 1,678 females, 1,338 males	To determine whether participants recognized the concept of a bucket list, and whether they were able to list specific items on their bucket list	Mixed methods; survey design	Identification of bucket lists; influence of age, gender, marital status, education level, and spirituality on the likelihood of having a bucket list	Provided insights into the types of life goals people prioritize
Chaves et al. (2016)	Positive interventions in seriously ill children: effects on well-being after granting a wish	–	78 children (5−18 years); Caucasian children; 45 children were males (57.7%). 38 fathers and 86 mothers were interviewed	To investigate whether granting wishes promotes positive psychological responses and positive physical changes (i.e., reductions in pain and nausea) in children with a life-threatening illness	Quantitative; randomized controlled study	Positive and Negative Emotional Style Scale; the Student Life Satisfaction Scale; the Brief Multidimensional Students’ Life Satisfaction Scale; the Pediatric Quality of Life Scale; the Benefit Finding Scale for Children; Beliefs in the Benevolence of the World Scale; the Youth Life Orientation Test; the Values in Action Inventory of Character Strengths for Youth; the Health-Related Pediatric Quality of Life Scale; the Center for Epidemiologic Studies-Depression Scale; medical status and perceived probability of survival at 1 and 5 years	Wish-granting promotes positive emotions, and its results last for several weeks
Darlington et al. (2013)	Granting wishes: parents’ perception of a wish fulfillment for a child with a life-threatening illness	Netherlands	235 parents, mean age = 44.5 years; parents of 68 girls (39.1%) and 106 boys (60.9%) participated in the study	To investigate the experience of children and parents during and after a wish is granted	Quantitative; survey design	N/A	The experience is a positive one with children experiencing more energy and parents being distracted from their child’s illness
Patel et al. (2018)	Impact of a Make-A-Wish experience on healthcare utilization	United States	568 children (48.6% female for both groups) with severe chronic illnesses	To evaluate the impact of receiving a wish from the Make-A-Wish Foundation on patient healthcare utilization and cost-saving benefit measures	Quantitative; randomized controlled study	Emergency department visits, urgent care visits; unplanned hospitalizations, associated length of stay, planned hospitalizations, and associated length of stay	Participation in the Make-A-Wish program may provide children with quality of life relief while reducing hospital visits and healthcare expenditures
Reeve et al. (2021)	Community implementation of the 3 Wishes Project: an observational study of a compassionate end-of-life care initiative for critically ill patients	Canada	101 dying patients, with a mean age of 68.1 years; 55.4% were female. Patients were predominantly White (*n* = 95, 94.1%); 5 (5.0%) patients were Indigenous	To evaluate the adaptability of the 3 Wishes Project to a community intensive care unit (ICU), and to describe the patients cared for with this palliative approach, as well as local implementation strategies	Quantitative; observational, descriptive study	Outcomes for this study included the type, timing, and cost of each wish, which person or service made and facilitated each wish, and if and why the wish was successfully facilitated or not	The 3 Wishes Project is a patient- and family-centered palliative care initiative, successfully adapted to this community hospital at relatively low cost
Schilling and Sarigiani (2014)	The impact of a wish: caregiver perceptions of the benefits of granted wishes for children with life-threatening illnesses and their families	United States	682 participants; mean age = 9.3 years; 54.9% male and 45.1% female. Children were predominantly Caucasian (*n* = 573), African American (*n* = 64), Hispanic (*n* = 10), American Indian/Alaskan Native (*n* = 4), Asian/Pacific Islander (*n* = 4), other ethnic backgrounds (*n* = 21), and 6 missing or non-reported ethnic backgrounds	The purpose of the investigation was to explore the perceived benefits of the wishes granted by the Make-A-Wish Foundation of Michigan	Mixed methods; survey design and interviews	N/A	The effects of wish fulfillment provide positive memories that endure for years and may provide a source of comfort for families
Shoshani et al. (2016)	The effects of the Make-A-Wish intervention on psychiatric symptoms and health-related quality of life of children with cancer: a randomised controlled trial	Israel	66 children with cancer, aged 5−12 (*M* = 10.39)	To evaluate the efficacy of the Make-A-Wish intervention for children with life-threatening cancer	Quantitative; a wait-list-controlled trial with 2 parallel groups	Psychiatric symptoms: The Brief Symptom Inventory 18; health-related quality of life: Pediatric Quality of Life Inventor; positive affectivity indicator: The Positive and Negative Affect Schedule for Children, The Life Orientation Test-Revised, and Herth Hope Index	The role of hope and positive emotions in fostering the well-being of children who suffer from serious illnesses
Takaoka et al. (2021)	Scale-up and sustainability of a personalized end-of-life care intervention: a longitudinal mixed-methods study	Canada	369 patients, age 68.1 years; most had medical admitting diagnoses; 182 were female, 322 were white	To describe how, over 6 years, the 3 Wishes Project was scaled up from a research project to become embedded as a widespread approach to end-of-life practice	Mixed-methods; longitudinal study and interviews	N/A	The 3 Wishes Program has demonstrated scalability and sustainability over 6 years, transitioning over 3 phases from a research project to a clinical program and to an approach to practice
Tedesco et al. (2020)	The Good Wishes Project: an end-of-life intervention for individuals experiencing homelessness	Toronto, Canada	20 participants; mean age = 59.3 years; 74.1% were male; all had a primary palliative diagnosis	To elicit provider perspectives on the utility of the GWP in the delivery of end-of-life care to a population of homeless and vulnerably housed individuals	Qualitative; semi-structured interviews	N/A	The wishes showcase the scarcity of resources this population has and offer a means to satisfy unmet basic needs
Ullrich et al. (2022)	What are the personal last wishes of people with a life-limiting illness? Findings from a longitudinal observational study in specialist palliative care	Hamburg, Germany	361 patients; mean age = 69.5 years; 49.4% were female	To explore the presence and common themes of last wishes over time and to identify factors associated with having a last wish	Mixed methods; longitudinal study, questionnaires, and interviews	Personal last wishes, distress thermometer, depression, and anxiety	In this late phase of their illness, many patients voiced their last wishes
Yamashita et al. (2017)	Unfinished business in families of terminally ill with cancer patients	Japan	642 patients and family; patient mean age = 74.06 years; 54.7% male patients; family mean age = 61.48 years; 64.6% female in family	The prevalence and types of unfinished business in families of end-of-life patients with cancer, explore depression and grief associated with unfinished business, and explore the factors affecting unfinished business	Quantitative; survey design	Unfinished Business; Depression and Grief: Patient Health Questionnaire, Brief Grief Questionnaire; Process of Preparedness for the Patient’s Death; Condition of the Family and Patient at PCU Admission; Degree of Involvement of Health Care Professionals During PCU Admission	Families with unfinished business have significantly higher depression and grief scores after bereavement compared with those without

### Data summarization

In concordance with the recommendations made by Arksey and O’Malley (2005) and PRISMA-ScR (Tricco et al. 2018), the data were summarized by presenting key findings related to the research questions. Thus, the findings were presented in 2 ways: (1) the production of tables and chart maps and (2) a narrative organizing the literature thematically as it relates to the domains of bucket lists or last wishes among terminally ill patients.

## Results

Of the 2,234 initial studies collected from the selected databases, 825 duplicates were removed by Covidence, 2 studies were removed manually, leaving 1,409 articles eligible for the title and abstract review. After completing the initial review, we excluded an additional 1,331 articles. Of the 76 articles that were eligible for full-text review, 65 records were deemed ineligible. As illustrated in Fig. 1, records were removed for the following reasons: no specific measure/indication for bucket-lists, wish fulfillment, or unfinished business or incorrect topic (*n* = 28), not a full-text article (*n* = 4), wrong patient population (*n* = 2), or non-scholarly article (*n* = 11). Thus, 11 articles were included in the final analysis. A summary of each selected study is presented in Table 1.

## Data analysis

Thematic content analysis was used to articulate identified themes among the key findings from each study (Anderson 2007). The authors noted all descriptions and findings from the included studies that were relevant to our research question and to the topic of bucket list and facilitation of wishes among palliative and hospice care patients. Themes and codes were iteratively re-examined by team members to ensure consistency in how they were applied across studies. While not all themes appeared in every study, the coding process ensured that when a theme was present, it was interpreted and labeled in a consistent manner. The thematic results have been documented in Table 2.

**Table 2.** Thematic results

Authors	Impact on holistic well-being	Role of family in wish fulfillment	Cultivation of gratitude	Collaborative leadership in wish fulfillment
Periyakoil et al. (2018)	x			
Chaves et al. (2016)	x		x	
Darlington et al. (2013)	x	x		
Patel et al. (2018)	x			
Reeve et al. (2021)		x		x
Schilling and Sarigiani (2014)	x	x	x	
Shoshani et al. (2016)	x			
Takaoka et al. (2021)				x
Tedesco et al. (2020)			x	
Ullrich et al. (2022)		x		x
Yamashita et al. (2017)		x		

## Findings

### Impact on holistic well-being

Among the 11 articles reviewed, 6 articles reported improvements in overall well-being through wish fulfillment in participants. Schilling and Sarigiani (2014) found that 69.6% of participants in their survey reported experiencing positive emotional reactions, and 35.4% of respondents indicated a reduction in stress, while 37.4% reported the creation of positive and enduring memories. In Periyakoil et al. (2018), the desire to accomplish personal goals and achieve life milestones was the topmost sought priorities, which reflects individuals’ need for purpose and an expression of hope. Shoshani et al. (2016) demonstrated that children who underwent wish-fulfillment interventions showed elevated levels of hope, positive emotions, and an enhanced health-related quality of life, with a decrease in anxiety and depression levels. Chaves et al. (2016) also reported a decrease in nausea, and increased feelings of gratitude and awareness of social support among children following wish fulfillment. Mothers of these children observed positive changes in their children’s quality of life across physical, emotional, and academic domains and reported reduced concerns about their children’s illnesses. Darlington et al. (2013) emphasized the immediate positive impact of wish fulfillment on children’s energy levels and the decrease of parental stress related to their child’s disease progression. Finally, Patel et al. (2019) found that wish recipients experienced a significant reduction in healthcare utilization, including fewer unplanned hospital admissions and emergency department visits, compared to those who did not receive wishes. This reduction resulted in substantial cost savings, underscoring the value of wish-fulfillment programs. Collectively, these findings illustrate the diverse advantages of wish-fulfillment interventions, which enhance emotional well-being, alleviate physical symptoms, and reinforce social connections.

### Role of family in wish fulfillment

Six studies highlighted the importance of strong familial connections, including effective communication and the resolution of issues, during the end-of-life process. Yamashita et al. (2017) reported that families with unresolved conflicts reported higher levels of grief and depression following bereavement. Enhancing familial relationships was frequently noted as essential for addressing these unresolved matters, thus impacting emotional well-being. Additionally, wish fulfillment was found to strengthen family bonds, resulting in cherished memories and fostering a sense of selflessness (Schilling and Sarigiani 2014). On the other hand, the 3 Wishes Project provided families with an opportunity to honor their loved ones while preserving their dignity, tailoring care to their needs, and facilitating a meaningful grieving process (Reeve et al. 2021). In Ullrich et al. (2022), the category named “Taking care of final matters” (e.g., “that the kids will make peace with each other”) reflects the importance of resolving familial conflicts before passing, which are central to emotional well-being. Darlington et al. (2013) highlighted the significant effect of wish fulfillment on families, with 92% of parents characterizing the experience positively and 33% comparing this event to major life milestones such as childbirth or weddings. Simultaneously, these interventions also carried emotional complexities, intertwining feelings of happiness and sadness associated with the child’s life-threatening condition (Darlington et al. 2013; Schilling and Sarigiani 2014). Again, end-of-life communication and discussions among family members emerged as a notable finding in this context. These insights illustrate the dual role of families as both sources of support and emotional complexity during the end-of-life experience.

### Cultivation of gratitude

The theme of fostering gratitude through wish-granting emerged as a prominent theme in wish-fulfillment interventions in 3 studies. Similarly, Schilling and Sarigiani (2014) highlighted that participants often expressed their intentions to support the Make-A-Wish Foundation, reflecting the positive impact of their own wish-fulfilling experiences. Chaves et al. (2016) found that children who engaged in the wish-fulfillment intervention reported heightened feelings of gratitude and hope in comparison to a control group. These children acknowledged the selflessness provided by others, which fostered a sense of thankfulness and love. Finally, Tedesco et al. (2020) illustrated the broader societal implications of wish-fulfillment programs through the Good Wishes Project, which supported individuals experiencing homelessness in end-of-life care. By encouraging wish fulfillment, care providers went beyond merely addressing medical needs, affirming the individual and dignity of their clients, and introducing a sense of celebration and worth that is often absent in their lives.

### Collaborative leadership in wish fulfillment

Healthcare professionals play a crucial role in facilitating personalized end-of-life care, as evidenced by three studies. Reeve et al. (2021) highlighted the role of bedside nurses in the 3 Wishes Project, where they enrolled and facilitated wishes for 75% of service users, demonstrating their vital contribution to patient-centered care. The initiative’s formalized structure provided a systematic approach to end-of-life practices, ensuring the consistent involvement of nurses in delivering compassionate care. Expanding on this, Takaoka et al. (2021) documented the evolution of the 3 Wishes Project from a research initiative to a sustainable clinical program. This transformation involved transferring leadership from research staff to bedside healthcare teams, resulting in an increase in service users’ enrolment and implementation of wishes over 6 years, all without incurring additional costs. Over time, this program became integrated into routine care practices, broadening its impact within organizational contexts and enhancing family involvement in care decisions. Trust and strong relationships between service users and healthcare professionals emerged as critical components of end-of-life care (Takaoka et al. 2021). In addition, Ullrich et al. (2022) found that individuals were more likely to have wishes at the onset of care, suggesting the need for institutionalized settings, which may create an environment of wish expression among the healthcare professionals. This finding highlights the dual significance of technical competence and empathetic connection in delivering care.

## Strengths and limitations

This scoping review provides a comprehensive synthesis of existing literature on end-of-life dream experiences and bucket list fulfillment among terminally ill individuals receiving hospice and/or palliative care. By systematically mapping the literature, this review highlights both the emotional and practical dimensions of wish fulfillment, positioning it as an important yet underexplored component of person-centered palliative care. We identified critical thematic domains, including the impact of wish fulfillment on holistic well-being, the role of family in end-of-life aspirations, and the significance of collaborative healthcare in facilitating these experiences. By synthesizing these findings, this review provides a strong foundation for future empirical research and policy development, particularly in integrating aspirations-based conversations into routine palliative care. Furthermore, the inclusion of diverse methodological (qualitative, quantitative, and mixed-methods studies) enhances the breadth of insights presented, allowing for a multidimensional understanding of the topic.

A key limitation in multiple studies is the retrospective nature of data collection, which may have introduced recall bias in the findings, as participants were asked to reflect on past experiences after bereavement (Takaoka et al. 2021; Ullrich et al. 2022; Yamashita et al. 2017). In some cases, caregiver accounts dated back nearly 20 years, further compounding recall bias and limiting the ability to fully capture the lived experiences of individuals in palliative care. Additionally, several studies assessed the benefits of wish fulfillment through caregivers, parents, or healthcare providers rather than directly from service users (Darlington et al. 2013; Schilling and Sarigiani 2014; Tedesco et al. 2020), potentially overlooking the personal significance of these experiences for those receiving care. Prospective, person-centered studies would provide a more nuanced understanding of how wish fulfillment impacts emotional well-being, decision-making, and overall quality of life.

Other methodological limitations affect the generalizability of findings. Periyakoil et al. (2018) restricted participation to individuals with internet access, English proficiency, and basic technological literacy, excluding those with limited digital access. The intervention in Chaves et al. (2016) showed statistically significant effects, but the relatively small effect size suggests it may not lead to substantial improvements in well-being. Many studies were also conducted within single institutions or specific healthcare settings, limiting external validity (Schilling and Sarigiani 2014; Takaoka et al. 2021). Furthermore, the overrepresentation of Caucasian participants in several studies (Chaves et al. 2016; Reeve et al. 2021; Schilling and Sarigiani 2014) reduces the applicability of findings to racially and ethnically diverse populations. Several studies also lacked validated or standardized measures, raising concerns about reliability and comparability across research (Takaoka et al. 2021; Yamashita et al. 2017). While this review systematically maps the existing literature, it does not fully assess the methodological quality of the studies included. The absence of formal quality appraisal means findings should be interpreted with caution, as some studies may have limitations in design, sample representativeness, or measurement validity. Future research should incorporate standardized assessment tools to evaluate the rigor of studies in this emerging field.

## Discussion

The findings of this scoping review underscore the transformative potential of wish-fulfillment interventions within palliative and hospice care. A key insight from this review is that engaging service users in end-of-life dream discussions leads to significant improvements in holistic well-being, encompassing psychological, emotional, and physical dimensions. Studies consistently highlight reductions in anxiety and depression, enhanced coping skills among caregivers, and the mitigation of distressing physical symptoms (Chaves et al. 2016; Darlington et al. 2013; Shoshani et al. 2016). These findings highlight the broader evidence from Ullrich et al. (2022) and Masterson et al. (2018), which emphasizes fulfilling or discussing the aspect of wishes or unfinished business as critical to reducing feelings of regret and enhancing emotional well-being. This focus on closure and emotional resolution underscores the therapeutic value of wish-fulfillment interventions as a component of holistic palliative care. This suggests that integrating bucket list discussions into routine palliative care could serve as a meaningful, service user-centered intervention that is consistent with the holistic goals of palliative care: to alleviate physical suffering while addressing emotional and psychological needs.

Importantly, the review also surfaced concerns about equitable access to wish-fulfillment interventions. Many of the studies reviewed did not include diverse populations or explore how social determinants of health, such as socioeconomic status, race, culture, or housing insecurity, may affect one’s ability to articulate or fulfill end-of-life wishes. Democratizing this aspect of palliative care requires intentional strategies to ensure that individuals from marginalized communities are not excluded from such interventions due to systemic barriers or resource constraints. Future work must attend to cultural meanings, feasibility, and perceived value of bucket list fulfillment in underrepresented populations. Furthermore, the findings suggest that fulfilling a bucket list item can foster gratitude among individuals receiving care. Previous research indicates that gratitude may enhance quality of life and act as a protective factor against psychological distress in palliative care settings (Althaus et al. 2018). This supports the argument that bucket list fulfillment could be a valuable end-of-life intervention.

Another key theme that emerged from this review is the vital role of family engagement in end-of-life wish fulfillment. Wish fulfillment has improved family relations with strengthening of emotional bonds and created cherished memories (Schilling and Sarigiani 2014). It extends beyond supporting the service user’s emotional needs and enhances familial cohesion, which can provide a sense of closure during the end-of-life. Open and collaborative family discussions about end-of-life preferences were pivotal in enabling meaningful conversations; however, challenges such as differing priorities between individuals and their loved ones highlight the need for structured approaches to facilitate these conversations. Future studies could also explore the impact bucket list fulfillment has on surviving family and loved ones’ bereavement outcomes.

Findings also suggest that healthcare professionals play a significant role in facilitating wish-fulfillment and end-of-life discussions. For example, members of the interdisciplinary teams were essential in implementing initiatives like the 3 Wishes Project, fostering service user advocacy, and ensuring the sustainability of compassionate care programs (Reeve et al. 2021; Takaoka et al. 2021). Effective communication between healthcare providers and families was essential, as participants often emphasized the need for trusted relationships with doctors and nurses (Lankarani-Fard et al. 2010). Despite the evident benefits of wish-fulfillment initiatives, several systemic barriers persist, including funding constraints, logistical challenges related to the service user’s declining health, and the need for greater interprofessional collaboration (Darlington et al. 2013; Schilling and Sarigiani 2014). These barriers are particularly acute for individuals facing economic hardship, cultural exclusion, or structural marginalization, further reinforcing the need for equity-focused program design. Addressing these barriers requires targeted policy initiatives, increased financial support, and the integration of culturally sensitive approaches to ensure equitable access to these interventions (Tedesco et al. 2020).

The concept of bucket lists or wish-granting has been explored in various care settings and populations. For instance, the 3 Wishes Project in Reeve et al. (2021) is a program implemented in the ICU by fulfilling personalized wishes that dignify service users, with creating memories for families, and fostering compassion in healthcare providers. This concept has become a replicable model of conserving care within hospital environments. Similarly, the Good Wishes Project in Tedesco et al. (2020) adapted this approach with people who are unsheltered and who are experiencing life-limiting illness in Toronto. The program focused on fulfilling wishes for a population often excluded from traditional models of palliative care and promoting person-centered care. These innovative models offer insight into how wish-fulfillment interventions can be adapted to serve populations traditionally left out of mainstream palliative care frameworks, but such programs remain exceptions rather than norms. Expanding access at scale will require cross-sector partnerships, intentional outreach, and resource allocation that prioritize equity. Future research could also explore the barriers and facilitators that influence palliative and hospice care providers in discussing bucket lists with service users.

The findings from this scoping review have several key implications for practice scholarship and policy. From a practical standpoint, integrating wish-fulfillment discussions into routine palliative care can significantly enhance service user-centered care by addressing psychological and emotional well-being. Healthcare professionals should receive training on how to facilitate these discussions in ways that align with service users’ values and preferences. Additionally, stronger partnerships with community organizations and wish-granting foundations could improve access to such interventions, ensuring that service users have opportunities to fulfill meaningful aspirations at the end of life. At the workplace level, palliative and hospice care institutions should consider developing formal protocols that incorporate service users’ wishes as core elements of advance care planning. Policies that foster interdisciplinary collaboration among social workers, nurses, and physicians can ensure that service users’ aspirations are meaningfully integrated into care plans. These findings resonate with the concept of organizational compassion, which emphasizes a system-level capacity to recognize, connect with, and respond to the suffering of service users (Frost et al., 2000). Facilitating bucket list conversations and experiences often requires coordination across disciplines, flexible institutional polices, and a shared ethos of person-centered care, all hallmarks of a compassionate organization. By embedding these practices within organizational structures, rather than relying solely on individual providers, healthcare settings can more effectively enable meaningful end-of-life experiences. Additionally, palliative and hospice care settings should work toward securing sustainable funding for structured wish-fulfillment programs to address existing accessibility gaps and further enhance the holistic nature of care in these settings.

## Conclusion, implications, and future direction

This scoping review signifies the compounding effect and benefits of wish fulfillment among terminally ill individuals, reinforcing its role as an essential component of holistic, person-centered end-of-life care. Despite the demonstrated value of wish-fulfillment interventions, gaps in research persist, particularly regarding cultural and socioeconomic disparities, long-term effects, and implementation challenges within the healthcare system. Healthcare providers should receive formal training and awareness on facilitating end-of-life conversations, including understanding the cultural nuances in last wishes, and ethical considerations in managing wish fulfillment. It was also found that there is a need for a clear ethical framework to determine how wishes are being granted, particularly in resource-limited settings. Different ways to embed these interventions could be through interprofessional education and reflective practice workshops, adapting current or creating new informal tools (e.g., prompts and conversation guides) that invite service users to share hopes and dreams without turning the tool into a rigid form or checklist, creating a space during rounds for staff to reflect on these concepts, and developing supportive organizational cultures that encourage creativity. Moreover, current programs may be inaccessible to individuals from low socioeconomic or minority backgrounds, which could contribute to creating disparities. Therefore, awareness and policies should be implemented, ensuring equitable access to these resources.

Future work and research could also explore the infusion of technology in wish fulfillment. For instance, integrating digital platforms or virtual reality could offer alternative ways to fulfill wishes, particularly for people with mobility limitations or other constraints. However, such approaches must be implemented with caution to ensure that technology does not overshadow the humanistic core of end-of-life care. It is essential that these tools enhance, rather than replace, compassionate interpersonal connection. Additionally, reliance on technology must not create new accessibility barriers for individuals who may lack digital literacy, resources, or the ability to meaningfully engage with these platforms. Research on technology-driven wish-fulfillment models could enhance accessibility in end-of-life care.

As palliative and end-of-life care continues to evolve, wish-fulfillment interventions represent a powerful resource in enhancing dignity and holistic experiences for terminally ill individuals. Thus, future research efforts could strengthen programs ensuring every individual is able to achieve a sense of peace, fulfillment, and closure during their care trajectory.
